# Microbial Metabolism of Levodopa as an Adjunct Therapeutic Target in Parkinson’s Disease

**DOI:** 10.3390/antiox15010120

**Published:** 2026-01-17

**Authors:** Jimmy B. Feix, Gang Cheng, Micael Hardy, Balaraman Kalyanaraman

**Affiliations:** 1 Department of Biophysics, Medical College of Wisconsin, 8701 Watertown Plank Road, Milwaukee, WI 53226, USA; 2 ICR (UMR 7273), CNRS, Aix-Marseille Université, 13013 Marseille, France

**Keywords:** microbiome, mitochondria-targeted drugs, levodopa, TPP^+^-based drugs, Parkinson’s disease

## Abstract

Parkinson’s disease is the second leading neurodegenerative disease of aging. For over five decades, oral levodopa has been used to manage the progressive motor deficits that are the hallmark of the disease. However, individual dose requirements are highly variable, and patients typically require increased levodopa dosage as the disease progresses, which can cause undesirable side effects. It has become increasingly apparent that the gut microbiome can have a major impact on the metabolism and efficacy of therapeutic drugs. In this Perspective, we examine recent studies highlighting the impact of metabolism by *Enterococcus faecalis*, a common commensal gut bacterium, on levodopa bioavailability. *E. faecalis* expresses a highly conserved tyrosine decarboxylase that promiscuously converts levodopa to dopamine in the gut, resulting in decreased neuronal uptake of levodopa and reduced dopamine formation in the brain. Mitochondria-targeted antioxidants conjugated to a triphenylphosphonium moiety have shown promise in transiently suppressing the growth of *E. faecalis* and decreasing microbial levodopa metabolism, providing an approach to modulating the microbiome that is less perturbing than conventional antibiotics. Thus, mitigating metabolism by the gut microbiota is an attractive therapeutic target to preserve and potentiate the efficacy of oral levodopa therapy in Parkinson’s disease.

## 1. Introduction

Parkinson’s disease (PD) is a progressive neurodegenerative disease resulting from the loss of dopaminergic neurons in the substantia nigra and a consequential deficiency of the neurotransmitter dopamine (DA) in the striatum [1]. PD is the second most prevalent neurodegenerative disease of aging, with an estimated seven to ten million cases worldwide [2]. The most recognized symptoms of PD are progressive movement disorders including tremors, stiffness, dyskinesia, and difficulties with balance and coordination. However, Parkinson’s patients also exhibit a variety of non-motor symptoms, including constipation, depression, hyposmia, and pain [2]. The incidence of PD has more than doubled in the past two decades [3,4], an accelerating burden that is consistent with an overall aging of the world’s population.

The etiology of PD is complex and not fully understood. Epidemiological studies indicate that non-familial forms of PD are age-related, with an incidence that increases steadily after age 65 [2,5]. Approximately 75–90% of PD cases are sporadic late-onset, with the remaining 10–25% due to genetic alterations [2,5]. Genetic causes of PD, although responsible for a minority of cases, do suggest potential pathogenic mechanisms, including impaired mitochondrial quality control, disruption or alteration of ubiquitination patters, and oxidative stress. Although the underlying causes of sporadic PD remain unknown, numerous lines of evidence support the involvement of oxidative stress and mitochondrial dysfunction in PD pathogenesis [6,7,8,9]. Aggregation of α-synuclein, a pathological hallmark of PD, impairs mitochondrial function and can interfere with mitochondrial autophagy [10,11]. Exposure to the neurotoxin MPTP (1-Methyl-4-phenyl-1,2,3,6-tetrahydropyridine), which causes Parkinsonism in humans and rodents, as well as other inhibitors of mitochondrial electron transport chain complex I, causes a loss of dopaminergic neurons in mouse models of PD [12]. In addition, a transgenic (‘MitoPark’) mouse model, generated by selectively knocking out mitochondrial transcription factor A in dopaminergic neurons, recapitulates many of the clinical features of PD including neuronal loss and progressive motor dysfunction [13]. Mechanisms for the production of reactive oxygen species (ROS) by mitochondria, strategies for scavenging mitochondrial ROS, and early studies on the use of antioxidants and mitochondria-targeted drugs (MTDs) as therapeutics in the context of PD have previously been reviewed [12].

Oral administration of levodopa (L-dopa) has been the front-line treatment for management of PD-associated movement disorders for over five decades [14,15,16,17]. Indeed, improvement in motor symptoms upon L-dopa treatment in the early stages of the disease is a primary indicator in PD diagnosis [2]. Orally administered L-dopa is absorbed into the bloodstream, crosses the blood–brain barrier, and is converted to DA in the brain, thereby helping to alleviate the debilitating motor symptoms that are the hallmark of the disease. There are, however, a number of caveats associated with L-dopa therapy in PD. These include cyclical relief from motor symptoms (“on-off” phenomena), high individual variability in effective dose, and a loss of efficacy over time [2]. Loss of efficacy over the course of the disease necessitates escalation of L-dopa dosage, elevating the risk of L-dopa-induced dyskinesia (LID). Development of LID occurs in up to 40% of PD patients receiving L-dopa therapy and increases with escalating L-dopa dose and duration of treatment [18,19,20]. The incidence of LID and other motor/non-motor fluctuations is increased in women [21], possibly due to higher serum levels of L-dopa at a given standard dose [21,22,23]. This suggests that personalization of L-dopa therapy to achieve the minimal dose required to alleviate symptoms may be an important goal in reducing the occurrence of LID [23], although this can be challenging in practice. Given the pre-eminent role of L-dopa therapy in managing PD symptoms, measures that enhance its efficacy or extend its useful lifetime while reducing the risk of LID are potentially of great benefit.

## 2. Gut Microbial Metabolism of L-Dopa

One important factor that could conceivably contribute to differences in patient response to L-dopa therapy and to the loss of L-dopa efficacy over time, which complicate personalization of treatment regimens, is individual variability in the gut microbiome. It has become increasingly apparent that the gut microbiome can have a major impact on the metabolism and efficacy of therapeutic drugs [24,25,26]. Numerous studies have found differences in gut microbial composition in PD as compared to healthy controls [27,28,29,30,31,32] and gastrointestinal symptoms, primarily constipation, are among the most common non-motor deficits associated with PD, often preceding clinical diagnosis by years [33,34]. Bacterial overgrowth in the upper region of the small intestine (jejunum), the primary site of L-dopa absorption [35,36], is observed in PD [37,38,39] and may contribute to metabolism of L-dopa prior to its reaching the bloodstream, thereby reducing its bioavailability. The incidence of small intestinal bacterial overgrowth (SIBO) is elevated in PD (48% as compared to 3% in age-matched controls), and L-dopa treatment efficacy is improved following eradication of SIBO by treatment with antibiotics [38]. In an alpha-synuclein overexpressing (ASO) mouse model of the disease, transplantation of intestinal microbes from PD patents exacerbated motor deficits [40], and this effect was also reversed with antibiotic treatment [40]. These observations all suggest a connection between the gut microbiota (or bacterial metabolites) and PD pathophysiology.

**Figure 1:**
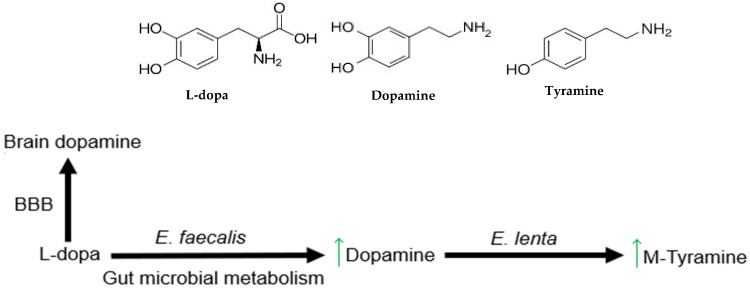
Gut microbial metabolism of L-dopa. L-dopa absorbed into the bloodstream crosses the blood–brain barrier (BBB) to increase dopamine in the brain. Gut metabolism by *E. faecalis* converts L-dopa to dopamine, effectively reducing the amount of L-dopa available for uptake. Dopamine produced in the gut can be further metabolized to m-tyramine by *E. lenta* (adapted with permission from [46]).

While the role of the gut–brain axis in the onset or progression of PD remains to be further explored, the impact of peripheral metabolism on oral L-dopa treatment has long been recognized [41,42] and there is now strong evidence that commensal bacteria present in the gut contribute to L-dopa degradation. Recent studies have shown that *Enterococcus faecalis*, a ubiquitous member of the gut microbiome, metabolizes L-dopa to DA [43,44], with further conversion of DA to tyramine by another commensal species, *Eggerthella lenta* [44] (Figure 1). Since DA produced systemically cannot cross the blood–brain barrier, these bacterial metabolic pathways effectively reduce L-dopa bioavailability. In addition, systemic DA has been associated with adverse physiological effects [17,45]. A genome-mining approach to search for homologs of a bacterial tyrosine decarboxylase (TyrDC) found in a food-associated strain of *Lactobacillus brevis* identified a number of hits among the enterococci [44]. When representative gut strains were tested in vitro, only *E. faecalis* showed complete decarboxylation of L-dopa under anaerobic conditions.

All *E. faecalis* strains tested were found to share a highly conserved operon encoding a TyrDC with very high sequence conservation, consistent with tyrosine decarboxylation being a common phenotypic trait of this species [44]. Similarly, a search based on the TyrDC from the clinical strain *E. faecalis* v583 confirmed conservation of the *tyrDC* operon among more than 50 *Enterococcus* strains, along with *Lactobacillus* and *Staphylococcus* species [43]. Comprehensive screening of clinical *Enterococcus* isolates confirmed their ability to metabolize L-dopa to DA [43]. Decarboxylation of L-dopa by purified *E. faecalis* TyrDC and by *E. faecalis* strains in culture was increased at lower pH, suggesting that L-dopa metabolism may be accelerated at the more acidic environment of the upper small intestine [43,44,47]. Mutagenesis and biochemical experiments demonstrated that TyrDC was both necessary and sufficient for L-dopa decarboxylation [44] and is exclusively responsible for L-dopa metabolism by *E. faecalis* [43].

L-dopa therapy is commonly accompanied by an inhibitor of peripheral eukaryotic L-amino acid decarboxylase (AADC) enzymes (e.g., carbidopa). However, carbidopa and related inhibitors were shown to be significantly less effective against purified bacterial TyrDC [43,44], and L-dopa metabolism by *E. faecalis* in batch culture was unaffected by the addition of up to 4-fold excess carbidopa [43]. Addition of carbidopa did not alter L-dopa decarboxylation or end-point tyramine production in stool samples from PD patients or neurologically healthy controls [44], consistent with an early report that carbidopa administration does not alter tyramine production in patients [48]. Thus, the bacterial tyrosine decarboxylases capable of metabolizing L-dopa in the gut are not effectively inhibited by carbidopa or other inhibitors of eukaryotic AADC.

Analysis of fecal samples from human subjects showed a strong linear correlation between *E. faecalis* abundance and abundance of the *tyrDC* gene, suggesting that *E. faecalis* is likely the dominant organism responsible for L-dopa decarboxylation [44]. Addition of *E. faecalis* to nonmetabolizing fecal samples led to complete consumption of L-dopa, confirming that *E. faecalis* could decarboxylate L-dopa in a complex community of gut microbes [44]. In PD patients, a higher relative abundance of the *tyrDC* gene in stool samples is associated with a higher daily L-dopa requirement and with duration of the disease [43]. In a relatively small study of 23 patients there was a strong inverse correlation between *tyrDC* gene levels and *E. faecalis* abundance and peak serum L-dopa concentration [36].

In contrast to the relative insensitivity of *E. faecalis* TyrDC to carbidopa [43,44], the bacterial enzyme was strongly inhibited by (S)-α-fluoromethyltyrosine (AFMT) [44]. AFMT reduced L-dopa metabolism by *E. faecalis*/*E. lenta* co-cultures and completely inhibited L-dopa decarboxylation by gut microbiota samples from PD patients or controls. In a model study utilizing gnotobiotic mice colonized with *E. faecalis*, AFMT treatment increased serum L-dopa levels following L-dopa/carbidopa administration [44]. These studies validate the concept that it may be possible to enhance the efficacy of L-dopa treatment by mitigating its metabolism by the gut microbiome.

## 3. MTDs—Antioxidant Effects and Inhibition of Microbial Metabolism

**Figure 2:**
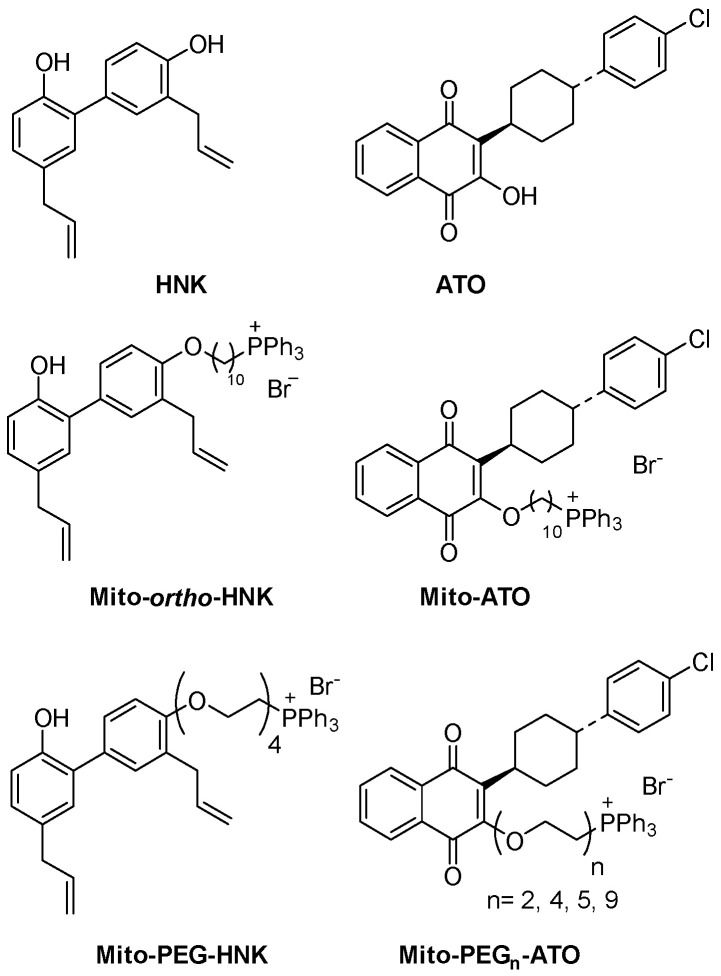
Chemical structures of parent compounds and their MTD analogs.

Given the potential roles of gut microbial dysbiosis and bacterial metabolism in PD pathology and treatment, we examined the effects of a series of novel antimicrobial compounds on *E. faecalis* growth and L-dopa metabolism [46]. These compounds (Figure 2) contain a triphenylphosphonium (TPP^+^) delocalized cation moiety and were initially designed as mitochondria-targeted drugs (MTDs) [49,50]. MTDs, prepared by conjugating TPP^+^ to the parent molecule via an alkyl linker, and can be fine-tuned for solubility and hydrophobicity by modulating the length of the alkyl chain or addition of a variable length polyethylene glycol (PEG) moiety [51,52]. MTDs have received considerable attention for the amelioration of oxidative stress in both in vitro and in vivo PD models [12]. For example, Mito-apocynin, a TPP^+^-derivatized analog of the plant-derived antioxidant apocynin, prevented mitochondrial dysfunction and neuronal damage in both 1-Methyl-4-phenyl-1,2,3,6-tetrahydropyridine (MPTP)-induced parkinsonism [53] and the transgenic MitoPark mouse model of PD [54].

In addition, Mitoquinone (Mito-Q), the most extensively studied MTD, has shown beneficial effects in a variety of in vitro and in vivo PD model studies [12]. Mito-Q protected against the loss of dopaminergic neurons in a cell culture model of PD and prevented dopaminergic neurodegeneration, preserved striatal dopamine, and improved motor functions in an MPTP mouse model [55]. Although a double-blind clinical trial failed to show any benefit from oral Mito-Q in slowing the clinical progression of PD [56], it may be that the extensive loss of dopaminergic neurons that occurs prior to PD diagnosis prevented observation of any benefit. Hence, further studies are needed to clarify the therapeutic effects of Mito-Q in PD subjects at an earlier stage of disease onset.

Bacteria, like mitochondria, have a large, inside-negative transmembrane potential that drives uptake and accumulation of MTDs [57], and some TPP^+^-derivatized compounds have been shown to have broad-spectrum antimicrobial activity [58,59,60]. We reasoned that if these compounds could be used to reduce *E. faecalis* abundance in the gut, they might improve L-dopa bioavailability. To investigate this principle, we chose to more closely examine antimicrobial activities for TPP^+^ derivatives of two compounds: honokiol (HNK), a natural product polyphenol that has demonstrated neuroprotective effects in mouse models of PD [61,62], and atovaquone (ATO), an analog of ubiquinone (coenzyme Q) that inhibits the mitochondrial cytochrome bc_1_ complex and has been approved for treatment of malaria caused by the parasites *Pneumocystis jirovecii* and *Plasmodium falciparium* [50,63]. Mito-*ortho*-HNK and Mito-PEG_n_-ATO analogs (Figure 2) were prepared containing variable length PEG chains to improve solubility and bioavailability [46]. Importantly, previous studies with these compounds in preclinical animal models demonstrated that they are well-tolerated without significant toxicity [49,50].

Mito-targeted analogs of HNK and ATO demonstrated good antimicrobial activity against a panel of Gram-positive bacteria, including *E. faecalis* [46]. Against Gram-positive strains, minimum inhibitory concentrations (MICs, the lowest concentration resulting in complete growth inhibition over 24 h) were in the 0.5–2 uM range (Table 1), comparable to MIC values for conventional antibiotics. For the Mito-PEG_n_-ATO compounds, MIC values against Gram-negative bacteria (e.g., *E. coli* and *Pseudomonas aeruginosa*) were 4 to 8-fold higher, demonstrating some degree of selectivity. Selectivity was even more pronounced for Mito-*ortho*-HNK, which showed strong activity against Gram-positive bacteria (MIC for *E. faecalis* of 2 μM) but was not active against Gram-negative bacteria up to 64 μM [46].

At concentrations below the MIC, Mito-*ortho*-HNK caused a dose-dependent delay in *E. faecalis* proliferation [46]. Following a delay of 2–6 h (depending on dose), *E. faecalis* proliferation recovered to grow at rates near that of untreated controls. Neither L-dopa nor carbidopa had any effect on *E. faecalis* in vitro growth rates. Coinciding with the delay in bacterial growth, Mito-*ortho*-HNK inhibited the metabolism of L-dopa by *E. faecalis*. In the presence of 3 μM Mito-*ortho*-HNK, L-dopa consumption and DA production after 4 h were inhibited by ~70%. Carbidopa had no effect on L-dopa metabolism by *E. faecalis*, consistent with it being a poor substrate for the bacterial tyrosine decarboxylase.

Comparable results were observed with PEGylated analogs of Mito-ATO (Mito-PEG_n_-ATO, n = 2, 4, 5, 9). Low concentrations of Mito-PEG_n_-ATO caused a dose-dependent delay in *E. faecalis* proliferation for a period of 2–4 h, followed by a return to normal growth kinetics [46]. Studies with Mito-PEG_5_-ATO showed a dose-dependent inhibition of L-dopa consumption and DA production, with 2 μM Mito-PEG_5_-ATO giving an inhibition of ~50% at the 2.5 h time point. In contrast to results with Mito-*ortho*-HNK and the Mito-PEG_n_-ATO compounds, the conventional antibiotics chloramphenicol and ampicillin produced dose-dependent growth inhibition but without subsequent recovery [46].

Membrane depolarization has been proposed as the mechanism of antibiotic action for the MTD, SkQ1 [59]. Mito-*ortho*-HNK caused a dose-dependent decrease in *E. faecalis* transmembrane potential, as indicated by decreased tetramethylrhodamine (TMR) fluorescence [46]. As controls, neither chloramphenicol nor ampicillin had any effect on bacterial membrane potential. Similarly, neither Mito-*ortho*-HNK nor the Mito-PEG_n_-ATO compounds caused overt membrane permeabilization, as indicated by SYTOX green uptake assays [46].

## 4. In Vivo Studies

**Figure 3:**
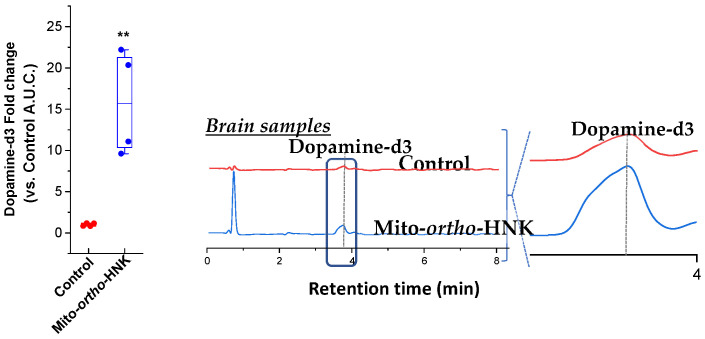
(**Left**) Fold increase in brain dopamine-d3 following administration of L-dopa-d3 in mice treated with Mito-*ortho*-HNK based on area under the curve (AUC) of HPLC curves. ** *p* < 0.01. (**Right**) Representative HPLC traces from brain tissue extracted and analyzed as previously described (Adapted with permission from [46]).

To assess the effects of Mito-*ortho*-HNK on L-dopa metabolism in vivo, mice were treated by oral gavage with isotopically labeled L-dopa-d3, either alone or in combination with Mito-*ortho*-HNK. The use of isotopically labeled L-dopa allowed its metabolism to DA to be followed, even in the presence of high concentrations of DA in the brain. Following tissue extraction, samples were monitored by HPLC-mass spectrometry with single ion monitoring (see [46] for detailed methods). In homogenized samples from the gut, Mito-*ortho*-HNK inhibited L-dopa-d3 consumption and the formation of dopamine-d3. In brain samples, a significant increase (~15-fold) in dopamine-d3 following L-dopa-d3 administration was observed for mice treated with Mito-*ortho*-HNK (Figure 3). These results indicated that Mito-*ortho*-HNK can inhibit L-dopa decarboxylation in the gut, thereby increasing the amount of L-dopa available for conversion to dopamine in the brain, over a time frame coincident with the transient inhibition of *E. faecalis* proliferation [46].

## 5. Conclusions

Despite the continuing development of new therapeutic strategies, oral levodopa therapy remains the gold standard for the treatment and management of PD symptoms. The peripheral metabolism of L-dopa, primarily by decarboxylation to DA, is an ongoing challenge that has been recognized since the introduction of L-dopa therapy more than fifty years ago. It is now recognized that bacterial metabolism of L-dopa in the gut may contribute substantially to its peripheral metabolism, and that inhibitors of eukaryotic AADC are relatively ineffective against bacterial TyrDC enzymes. Only recently has it been demonstrated that *E. faecalis*, a common commensal Gram-positive bacterium that is ubiquitous in the mammalian intestinal flora, is primarily responsible for the microbial metabolism of L-dopa [43,44]. Our recent studies suggest that MTDs may be useful to transiently inhibit *E. faecalis* metabolism and the associated decarboxylation of L-dopa without causing substantial perturbation of the gut microbiome. Given the antioxidant properties of many MTDs, there is a potential for synergistic neuroprotective benefits that remains to be explored. Although tetracycline antibiotics (doxycycline, minocycline) have shown neuroprotective effects in mouse models of PD [64,65], treatment with conventional antibiotics can seriously disrupt the normal microbial flora, enabling proliferation of drug-resistant, pathogenic bacteria [66,67,68]. Thus, transient inhibition of *E. faecalis* growth is a novel strategy to inhibit L-dopa metabolism during the period of intestinal absorption without causing a significant perturbation of the normal gut microbiome, thus increasing the amount of L-dopa available for uptake and conversion to DA in the brain. Additional neuroprotective benefits may be realized by developing novel molecules that combine antimicrobial and antioxidant activities. The development of adjutant compounds that preserve the efficacy of L-dopa therapy over the duration of the disease may provide substantial benefits for the millions of patients that rely daily on L-dopa for the management of symptoms associated with PD.

## Figures and Tables

**Table 1:** Effect of mitochondria-targeted analogs on minimal inhibitory concentrations in Gram-negative and -positive bacteria (adapted with permission from [46]).

	Minimal Inhibitory Concentrations (μM)					
	*S. aureus*	*B. subtilis*	*E. faecium*	*E. faecalis*	*E. coli* BL21	*P. aerug* PA01
HNK	32	64	64	64	NA	NA
Mito-*ortho*-HNK	1	1	0.5	2	NA	NA
Decyl-HNK	NA	NA	NA	NA	16	32
Mito_10_-ATO	16	4	8	8	64	64
Mito-PEG_2_-ATO	1	1	1	1	4	8
Mito-PEG_4_-ATO	1	1	1	1	4	8
Mito-PEG_5_-ATO	1	1	1	1	8	16
Mito-PEG_9_-ATO	2	2	2	4	8	16

NA = not active. No inhibition of cell growth at 64 µM.

## Data Availability

No new data were created or analyzed in this study. Data sharing is not applicable to this article.
